# Variability of Renal Ultrasound Measurements: How Physician Experience and Patient Position Affect Measurement Accuracy?

**DOI:** 10.3390/jcm14165840

**Published:** 2025-08-18

**Authors:** Dominik Świętoń, Gabriela Hryniewicz, Małgorzata Grzywińska, Mariusz Kaszubowski, Wojciech Kosiak, Piotr Czarniak, Joanna Świętoń, Hanna Storoniak, Maciej Piskunowicz

**Affiliations:** 1 2nd Department of Radiology, Medical University of Gdansk, 80-214 Gdansk, Poland; 2 University Clinical Center, Medical University of Gdansk, 80-214 Gdansk, Poland; 3 Department of Neurophysiology, Neuropsychology and Neuroinformatics, Medical University of Gdansk, 80-210 Gdansk, Poland; 4 Department of Statistics and Econometrics, Gdansk University of Technology, 80-211 Gdansk, Poland; 5 Department of Pediatric, Hematology & Oncology Department, Medical University of Gdansk, 80-952 Gdansk, Poland; 6 Department of Pediatrics, Nephrology and Hypertension, Medical University of Gdansk, 80-952 Gdansk, Poland; 7 Department of Nephrology, Transplantology and Internal Medicine, Faculty of Medicine, Medical University of Gdańsk, 80-952 Gdansk, Poland; 8 1st Department of Radiology, University Clinical Center, Medical University of Gdansk, 80-214 Gdansk, Poland

**Keywords:** kidney, ultrasonography, patient positioning

## Abstract

This study was designed to investigate the variability of renal ultrasound measurements, focusing on the impact of physician experience and patient position. **Background**: Since decreased kidney length is considered an indicator for chronic renal disease, understanding measurement repeatability and reproducibility is crucial for establishing effective diagnostic guidelines. **Methods**: Fifty healthy young adults underwent renal ultrasound scans performed by three examiners with varying levels of experience (12 years, 5 years, and 4 weeks). Renal length was measured at the level of the hilum in three patient positions: supine, lateral decubitus, and prone, using a 2–6 MHz convex probe (GE Logiq S8). **Results:** This study found that examiner experience significantly affected the results of sonographic measurements. However, the Interclass Correlation Coefficient analysis for all examiners demonstrated good reliability in most positions, with the highest values observed for the prone position. Measurements in the lateral decubitus position showed highest values, especially for the most experience examiner. The less experienced sonographers produced more variable results. **Conclusions:** Standardized patient positioning improves the accuracy and reproducibility of renal ultrasound measurements. The prone position offers a balance of reliability and practicality, especially for less experienced operators.

## 1. Introduction

Ultrasonography (US) plays an important role in diagnosing and evaluation kidney diseases. The advantages of US, such as clinical utility, accessibility, and non-invasive character, make this imaging modality the method of choice in nephrology and urology [1,2]. The basic parameter of the urinary tract in US is renal size assessed in three dimensions, often complemented by calculated renal volume. A reduced renal size is often seen as an indication of both congenital and acquired renal abnormalities. Renal length and volume are highly relevant parameters, being indirect indicators of renal function. Renal volume correlates with glomerular filtration rate; it is considered the best parameter of kidneys. However, renal length correlating with renal volume is the most clinically useful US parameter [3,4].

Renal dimensions are utilized for both diagnostic and prognostic purposes, with therapeutic decisions frequently guided by kidney size and morphology. The measurements should be taken precisely and in a repeatable manner; otherwise, interpretating the ultrasonography results can be challenging, potentially leading to misdiagnosis [5].

The kidneys are located in the retroperitoneal space. The left kidney is positioned between the T12 and L3 vertebral levels and is usually 1 to 2 cm higher than the right kidney. The anatomic differences have an impact on imaging results, especially in ultrasound.

There is a limited number of studies analyzing the accuracy of US renal size assessments [4,6,7]. The focus of these studies has typically been the comparison between sonographic measurements and other imaging techniques. It is often overlooked that US is a subjective method, and the results rely significantly on the experience of the examiner. The aim of this study is to assess the reliability of renal measurements in relation to the examiner’s experience and the impact of body position on the results in a population of young adults.

## 2. Materials and Methods

This prospective study was designed to evaluate the variability of renal ultrasound measurements depending on the examiner’s experience and patient position.

### 2.1. Participants

The examined group included fifty healthy young adults, who were students at Gdansk Medical University, Gdansk, Poland. The group consisted of 27 females and 23 males aged 20 to 23 years, with no history of kidney disease. The body mass index (BMI) of the participants ranged from 18.5 to 29.9. Exclusion criteria included anatomical anomalies. Consequently, three participants were excluded from the study: two due to duplex kidney and one due to right renal agenesis, resulting in a study size of 47 individuals. The inclusion criteria—normal body habitus, absence of kidney disease, and specific age range—required a highly selective population. This was intentional, ensuring methodological consistency and minimizing distractions for examiners.

### 2.2. Ultrasound Equipment and Settings

All examinations were conducted using a GE Logiq S8 ultrasound device (GE Medical Systems, Milwaukee, WI, USA) equipped with a 2–6 MHz convex probe. The ultrasound settings were standardized as follows: the depth was adjusted to encompass the entire kidney, the focus was placed below the lower outline of the kidney, and the frequency and gain were optimized for the best visualization of the kidney margins.

### 2.3. Examiners and Measurement Protocol

The ultrasound examinations were carried out by three sonographers with varying experience in renal ultrasonography: Examiner 1, a specialist with 12 years of experience in renal ultrasonography, Examiner 2, a radiology resident with 5 years of experience in renal ultrasonography, and Examiner 3, a student at Medical University in their final year after four weeks of training.

Each examiner independently measured the maximum length, width, and thickness of both kidneys (left and right) for each participant. Measurements were taken in three different patient positions: supine, prone, and lateral decubitus position (LD position). Measurements for each position included the following: kidney length (measured in the longitudinal plane as the maximum distance between the upper and lower poles of the kidney), kidney width, and thickness (measured in the transverse plane at the level of the renal hilum). Width was defined as the maximum dimension perpendicular to the longitudinal axis, while thickness was measured perpendicular to both length and width.

Understanding the course of the kidney’s long axis is crucial for accurate renal measurements; however, this information is most reliably obtained through a lateral approach. The long axis runs parallel to the lateral border of the psoas muscle, resulting in an oblique orientation where the superior pole is positioned more medially and posteriorly than the inferior pole.

The examination began with the patient in the supine position. The long axis of the kidney was identified with the probe placed along the anterior axillary line. Then, the patient was asked to turn into the lateral decubitus position, with the probe positioned along the mid-axillary line. Finally, the kidney was measured in the prone position, with the probe located along the posterior axillary line.

Measurements were recorded on freeze images, ensuring the kidney was visualized at its maximum dimension. Each measurement was performed once for each kidney in each position by each examiner.

The volume of the kidneys was calculated according to the formula for an ellipsoid:

Volume = Length × Width × Thickness × $\frac{\pi }{6}$, where Length, Width, and Thickness corresponded to the measured dimensions of the kidney.

### 2.4. Examination and Data Collection

To minimize the risk of systematic error during this study, we used a blinding procedure for the operators. The examiners did not have access to previous measurement results of other examiners or to previous data of the patients. All examinations for one participant were conducted on the same day, with examiners performing measurements sequentially to maintain uniform examination conditions. The examination was organized such that each examiner completed all measurements for a given participant in all three positions before proceeding to the next participant.

Measurement data were recorded and stored in a system that prevented examiners from accessing others’ results. Data analysis was conducted by an independent researcher who did not participate in the ultrasound examinations and was unaware of the examiners’ identities.

### 2.5. Statistical Analysis

We conducted a post hoc analysis using G*Power 3. The results showed that with 50 participants, a repeated measure ANOVA (f = 0.25, α = 0.05, r = 0.7) achieved a power of 0.999, indicating excellent sensitivity to detect a medium effect. These findings confirm that our sample size is sufficient to ensure the reliability of the study.

For the statistical analysis, the software SPSS Statistics 25 (IBM, Armonk, NY, USA), Microsoft Excel 2016 (Microsoft Corporation, Redmond, WA, USA), and Python 3.13 (Python Software Foundation) with the matplotlib library were. Descriptive statistics, including mean values and standard deviations, were calculated for kidney length and volume for both examiner and patient positions. To evaluate differences in measurements between examiners, a repeated measures analysis of variance (ANOVA) was applied. To examine the reliability and agreement between examiners for each patient position, the Interclass Correlation Coefficient (ICC) was analyzed. Statistical significance was set at *p* < 0.001. ICC values were interpreted using the following thresholds: poor agreement (ICC < 0.40), fair (ICC = 0.40–0.59), good (ICC = 0.60–0.74), and excellent (ICC = 0.75–1.0).

To complement the statistical analyses, heatmaps were generated using Python to visualize the Percentage Absolute Differences (PADs) in measurements across examiners and positions. The PAD was calculated as the absolute difference between two measurements divided by their mean, expressed as a percentage. Additionally, box plots were employed to display the distribution of kidney length and volume measurements, including medians, interquartile ranges, and potential outliers. Basic assumptions for parametric testing, such as normality and homogeneity of variance, were also assessed.

## 3. Results

### 3.1. Examiner Experience and Measurement Variability

**Figure 1:**
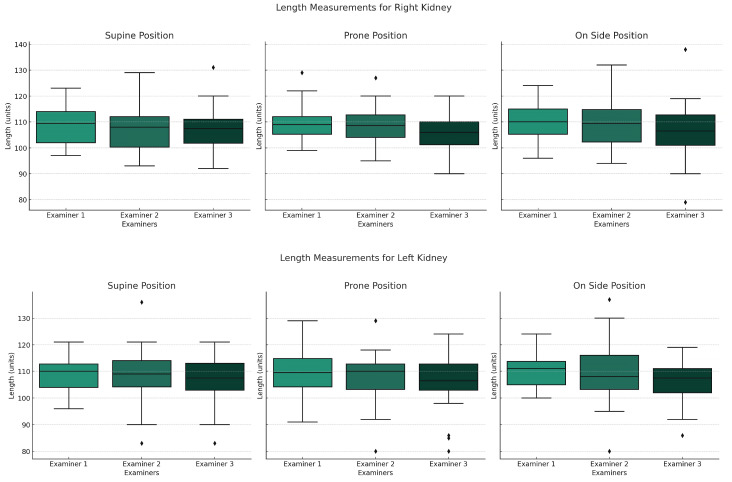
Graphs presenting distribution of renal measurements depending on body position for each examiner. Boxplots show the median (horizontal line), interquartile range (box), and whiskers extending to the most extreme values within 1.5 times the interquartile range. Dots indicate outliers.

All examiners were analyzed separately; none was treated as the reference. The distribution of renal measurement results and the differences between examiners in relation to body position are presented in Figure 1. Examiner 1 has the highest average results, followed by Examiner 2 and finally Examiner 3. Examiner 3 tends to underestimate kidney measurements in most positions.

**Figure 2:**
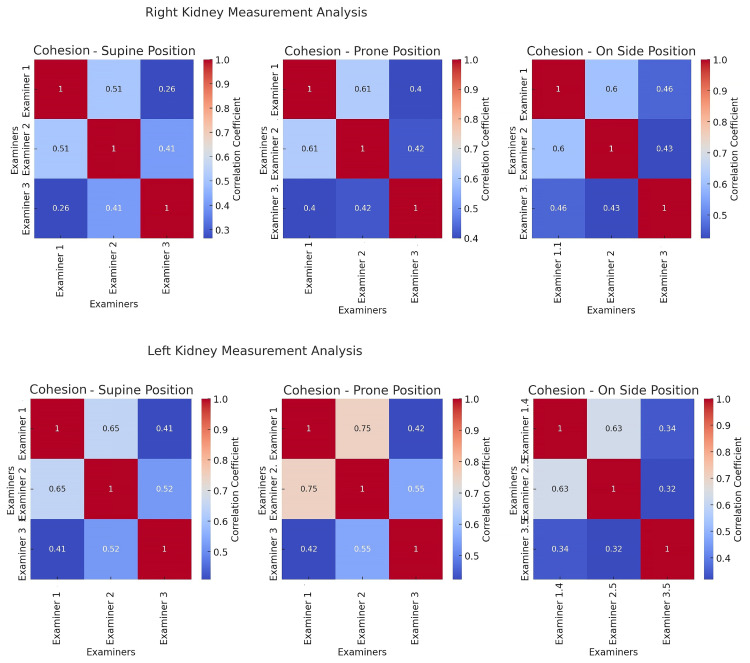
Cohesion heatmaps. These heatmaps display the correlation coefficients between measurements taken by different examiners for each position. A higher correlation coefficient indicates a stronger agreement in measurement patterns between the examiners.

The results differed between examiners but were cohesive (*p* < 0.001). The highest cohesion values were observed in prone position, ranging from 0.4 to 0.75. The cohesion was significantly higher between Examiner 1 and 2, and not exceeding 0.55 for Examiner 3 (Figure 2).

### 3.2. Influence of Patient Positioning

The highest values of renal length and volume were observed in the lateral decubitus position for Examiner 1 and 2. However, in the lateral decubitus position, there was a statistically significant difference between the third examiner and the more experienced examiners. For all examinations mean values are listed in (Table 1).

The Interclass Correlation Coefficient (ICC) analysis revealed good reliability across most positions, with the highest values in the prone position (Table 2). However, the cohesion between Examiners 1 and 2 exceeded that involving Examiner 3, rarely surpassing 0.55 for the latter (Figure 1).

Despite the challenges in assessing cohesion in this study, we believe that the collected data are reliable and reflect reality.

**Figure 3:**
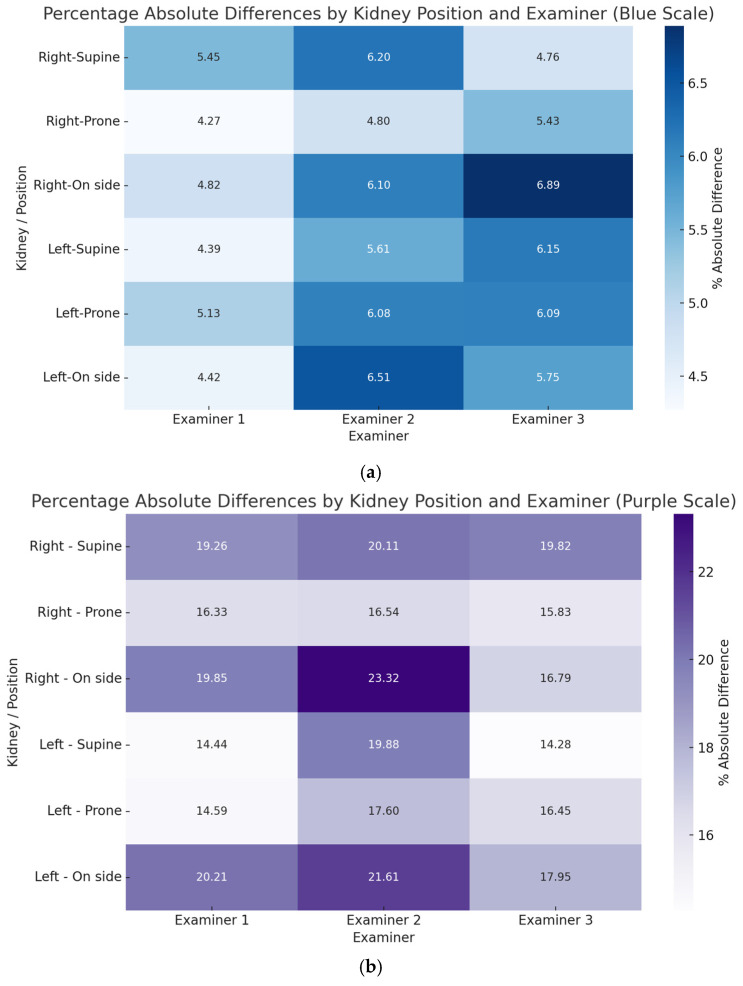
Heatmaps show the Percentage Absolute Differences (PADs) values across different positions for each examiner and kidney, visually comparing variability (**a**) for the kidney’s length and (**b**) for the kidneys volume.

The Percentage Absolute Differences (PADs) heatmaps (Figure 3) highlight the prone position as having the lowest variability in both renal length and volume, suggesting it as a potential candidate for standardization. The heatmaps present percentage of absolute differences. The lateral decubitus position was burdened with the highest differences, while results in the prone position were comparable between examiners. The lowest differences between body positions were observed for the Examiner 1. When analyzing kidney volume, the lowest differences were observed in the prone position for all examiners.

## 4. Discussion

Chronic kidney disease (CKD) is often described as one of most neglected and underdiagnosed chronic diseases. Both economic and epidemiological data are alarming, highlighting the need for early detection. In 2024, CKD was recognized as the fifth leading cause of years of life lost (YLL) [8]. CKD, particularly in the G5 stage, generates a significant burden for healthcare systems worldwide, with the cost of transplant in first year reaching approximately USD 75,326 [9].

Kidney length and volume are commonly accepted indicators of renal diseases, often forming the basis for decisions concerning renal disease [10]. Renal parenchymal thickness is also related to estimated glomerular filtration rate (eGFR) and has prognostic value in cases of renal impairment [11]. However, previous studies comparing the reliability of renal parenchymal thickness and its clinical implications have presented inconsistent results. Braconnier et al. (2021) [7] reported only a weak correlation between ultrasound (US) and magnetic resonance imaging (MRI) measurements of parenchymal thickness. Yet, Hoi et al. (2018) [12] strongly suggested that parenchymal thickness has predictive value for chronic kidney disease (CKD). These inconsistent results make it difficult to determine how effectively renal parenchyma thickness measurements can be used in clinical practice.

Ultrasonography appears as a cornerstone in the evaluation of renal pathology due to its non-invasive nature, widespread availability, and cost-effectiveness. The reliability of renal measurements performed during ultrasound examinations has been established in various studies comparing ultrasound findings with those obtained from other imaging techniques, including MRI and CT [7,13,14].

However, the method is known to be error-prone, stemming from operator-dependent factors, which limit its reliability as a diagnostic and prognostic tool [15,16]. The two-dimensional nature of ultrasound possesses limitations, especially when assessing renal volume; the ellipsoid or equivalent formulas that have been used carry a risk of underestimation. Taking this specific parameter into account, magnetic resonance imaging (MRI), especially with the voxel-count method, can provide more reliable results.

This study has several limitations. First, we did not account for anatomical variability and its influence on length measurements. Second, the study did not differentiate the group of patients according to body habitus. The primary focus of this study was to investigate two critical variables influencing the accuracy of renal ultrasound measurements: examiner experience and patient positioning. Thirdly, the study is limited by the relatively small number of participants. Despite listed limitations, our findings align with the existing literature while offering new insights into optimizing renal US protocols, particularly in a controlled cohort of healthy young adults [16,17]. The results clearly demonstrate a dependence on measurements accuracy related to examiner experience. Examiner 1, with 12 years of experience, consistently reported the highest mean renal length and volume values, followed by Examiner 2 (5 years), with Examiner 3 (a final year medical student) tending to underestimate these parameters. Examiner 1’s higher average values may be associated with their technical proficiency. This tendency stems from their consistent approach to acquiring the longest possible measurement, thereby reflecting the actual renal length. This trend underscores operator expertise as an important factor in the subjectivity of US.

Tolsgaard et al. (2013) [18] conducted a multi-center study, using Delphi technique, that resulted in the development of the Objective Structured Assessment of Ultrasound Skills (OSAUS). The scale aimed at evaluating ultra-sonographers’ skills across all fields of medicine. Unfortunately, this study does not specify reliable measurement assessment as part of the training process. Even though the authors acknowledged that determining the moment of proficiency in ultrasonography is inherently difficult, emphasizing the importance of considering experience when evaluating competency, they do not include the number of conducted studies as a part of training. There remains room for discussion and development of tools that enable systematic supervision of ultrasound examinations [18].

The statistically significant differences in lateral decubitus measurements between Examiner 1 and Examiner 3 (*p* < 0.001) suggest that less experienced sonographers may struggle with technical problems in more demanding examination conditions. This observation is consistent with prior studies, such as Ablet and Coulthard (1995) [19], where the authors emphasize intra- and inter-observer variability as a limiting factor in US reliability. These findings challenge the radiological community to address how much variability is acceptable in clinical practice and whether standardized training can further enhance interobserver agreement.

Patient position emerged as a significant factor in the outcomes of renal measurements [16,17]. The highest renal length and volume values were obtained in the lateral decubitus position by Examiners 1 and 2, which is in accordance with Jang et al. (2016) [20], who proved strong correlation of measurements in this position and Magnetic Resonance-derived renal dimensions.

However, the lateral decubitus position also exhibited the greatest interobserver variability, particularly with Examiner 3, indicating its technical complexity and reliance on experience. In contrast, the results in the prone and supine positions were the most cohesive across all examiners (ICC ranging from 0.60 to 0.75), suggesting these positions offer greater reproducibility. This is corroborated by Kouba et al. (2016) [21], who noted stable renal dimensions in prone positioning, particularly in pediatric populations.

While the exact mechanism is unclear, the lateral decubitus position may allow for better alignment of the ultrasound beam with the long axis of the kidney, potentially minimizing interference such as reflections from the gastrointestinal tract and ribs. Adopting the lateral decubitus position for renal examinations may improve diagnostic concordance with gold-standard modalities. However, this must be balanced against its higher variability among less experienced operators. The examination of a patient in the lateral decubitus position may be prone to errors when assessing the left kidney. The left kidney is located below the spleen, surrounded by ribs, and overlain by bowel gas. However, it is thought that when the ultrasonographic probe is positioned in the lateral decubitus stance, it aligns most closely with the true long axis of the kidney [16]. The prone position, while less reflective of true renal size compared to advanced imaging, offers a practical compromise due to its reproducibility.

This study examines only a population of young adults. The less experienced sonographers encounter more complex challenges then simply identifying the best imaging window or measuring the largest dimension of an organ—for example, working with geriatric or obese patients. Obesity becomes one of those most significant challenges in modern medicine. Questioning the effect of adipose tissue on the reliability of ultrasound imaging has become a relevant issue. To date, no studies have examined the variability of ultrasound images in obese adults while considering the experience level of the physician.

It is crucial to recognize that additional research is needed to better understand the reliability of ultrasound across diverse populations and to evaluate how the experience level of sonographers impacts the quality of imaging outcomes. We would like to emphasize the importance of ongoing efforts to improve patient safety by minimizing diagnostic inaccuracies.

## Figures and Tables

**Table 1:** The mean values of renal length and volume for each examiner.

Kidney			Descriptive Statistics					
Measure	Side	Position	Examiner 1		Examiner 2		Examiner 3	
			Mean	SD	Mean	SD	Mean	SD
Length	Right	Supine	108.5	6.827	107.2	8.37	107.2	6.83
		Prone	109.3	6.104	108.2	6.54	105.6	7.21
		Lateral decubitus	110.4	6.431	108.9	8.54	106.9	9.70
	Left	Supine	109.2	5.930	109.1	8.45	107.1	8.39
		Prone	109.7	7.358	108.2	8.87	106.7	8.82
		Lateral decubitus	110.6	6.199	109.3	9.59	106.4	7.56
Volume	Right	Supine	128.8	34.048	125.5	30.20	130.1	33.44
		Prone	122.1	24.127	111.4	22.05	113.1	23.17
		Lateral decubitus	144.0	34.566	132.1	39.90	116.2	27.35
	Left	Supine	135.8	27.656	137.7	36.13	129.8	22.79
		Prone	126.3	22.939	120.1	25.53	120.5	24.45
		Lateral decubitus	156.324	40.183	145.0	37.29	132.3	30.87

**Table 2:** The absolute compatibility and cohesion results in relation to body position.

Kidney			Absolute Compatibility			Cohesion		
Measure	Side	Position	ICC(2.3)	CI (95%)	*p*-Value	ICC(2.3)	CI (95%)	*p*-Value
Length	Right	Supine	0.67	0.45–0.80	<0.001	0.66	0.45–0.80	<0.001
		Prone	0.70	0.51–0.82	<0.001	0.73	0.55–0.84	<0.001
		Lateral decubitus	0.71	0.53–0.83	<0.001	0.72	0.55–0.84	<0.001
	Left	Supine	0.75	0.59–0.85	<0.001	0.76	0.60–0.86	<0.001
		Prone	0.79	0.66–0.88	<0.001	0.80	0.67–0.88	<0.001
		Lateral decubitus	0.65	0.44–0.79	<0.001	0.67	0.47–0.81	<0.001
Volume	Right	Supine	0.65	0.46–0.80	<0.001	0.65	0.46–0.80	<0.001
		Prone	0.66	0.50–0.82	<0.001	0.68	0.53–0.83	<0.001
		Lateral decubitus	0.60	0.32–0.75	<0.001	0.65	0.38–0.77	<0.001
	Left	Supine	0.71	0.23–0.72	<0.001	0.71	0.24–0.72	<0.001
		Prone	0.75	0.66–0.87	<0.001	0.76	0.66–0.88	<0.001
		Lateral decubitus	0.66	0.40–0.78	<0.001	0.70	0.47–0.81	<0.001

## Data Availability

All necessary tables and figures for understanding and evaluating the study are included in the manuscript.

## Abbreviations

The following abbreviations are used in this manuscript:

US	Ultrasonography
ICC	Interclass Correlation Coefficient
LD	Lateral decubitus
YLL	Years of life lost
CKD	Chronic kidney disease
PAD	Percentage Absolute Difference
